# The Recuperative Power of Old Age

**Published:** 1886-09

**Authors:** H. Boyle Runnalls

**Affiliations:** Saltash


### THE RECUPERATIVE POWER OF OLD AGE.

BY

H. Boyle Runnalls, M.R.C.S.,

*Saltash.*

---

It appears to be the accepted opinion of the public, and of our profession more or less, that old age possesses little recovering power; but, from observation, I am led to think that advanced age is not such an important negative factor in the prognosis of wounds, injuries, operations, or disease as it is stated to be. When we remember that in old persons the physical powers are becoming weakened; that the wear and tear is not so easily combated; that the skin and internal organisms are lessening in their secretory powers; that the muscular tissue is becoming atrophied, or undergoing fatty degeneration, from want of use; that the brain and spinal system are losing their power; and that the circulatory system is undergoing calcareous degeneration,—it does not surprise one that old age is not given credit for much perseverance in repairing wounds or struggling with disease. Actions speak more than words, which is in this case proved by the following records, and that must be my apology for infringing upon the space of this valuable *Journal*. It is always well to remember that "many mickles make a muckle."

James Deacon, Cargreen, age 71. At the age of 30 he received a kick on the back part of his right leg, which was followed by an abscess. This continued to discharge for several years, when it was found that he had necrosis of the lower end of femur. He was advised to have it removed, but would never consent to its being done; preferring to suffer the pain, rather than run the risk of an operation. It completely incapacitated him from following his occupation, and he spent the greater portion of his time in bed, owing to the pain which was produced by walking. He came under my notice within a few days of his seventy-first birthday, in 1882. I advised him to run the risk of an operation, as I thought if it could be performed without much loss of blood, there was no reason why he should not recover; for the healing process would not produce so much pain and discharge as the disease itself did. After-events proved me to be right. I removed the thigh at the lower third, making "Teale" flaps. At the end of a month the flaps had united; and at the termination of five weeks he was able to get about on crutches. He is still living, and has gained considerably in weight since the operation was performed.

John Hosking, Landulph, age 86. When this man first came under my notice he was suffering from apoplexy, from which he made a good recovery. Ten days after the hæmorrhage had taken place, a small red spot appeared over the front of lower third of tibia. He complained of great pain in it. This enlarged in size until it was about four inches in length and three inches in breadth. This after a few days became gangrenous, with the formation of a slough, extending down to the periosteum, and laying bare the anterior surface of the tendon of the tibialis anticus muscle for about three inches of its length. Within a short time I found that the anterior half of the tendon was necrosed; and it was particularly interesting to watch, day by day for about six weeks, the granulations slowly encircling the healthy portion and forming a sheath for it, and gradually throwing off the necrosed portion. After this became detached the granulating process made rapid strides, and in a few weeks the wound was filled, and soon became covered over with skin. He is now able to get about, and enjoys good health.

Elizabeth Hambly, Saltash, age 70. Had consolidation of both lungs. Made good recovery, and is now well and strong.

W. R., age 73. Had consolidation of both lungs. Made good recovery.

W. W., age 86. Had broncho-pneumonia. Made good recovery at end of five weeks.

M. C., age 89. Had fracture of patella. Made good union. She now walks well, the accident happening two years since.

W. H., age 72. Had fracture of patella eighteen months ago, followed by broncho-pneumonia. Is now able to walk well, good union having taken place.

I well remember a case which came under my notice in St. Marylebone Workhouse. It was that of a man, age 89. He was completely paralysed. He was attacked by small-pox, but made a good recovery.

I have selected these cases because of their severity, and as they necessarily more forcibly portray the restorative power which many old persons have. The operation for cataract is a good evidence of the store of repairing power which old age has. I consider that the case of James Deacon is rather remarkable; for all the surgical authorities that I have read state that the operation for amputation of the thigh, after the age of fifty, is almost certain to be fatal. This proves the opposite. No doubt the long continuance of the disease, and the operation being performed in a country cottage, were the two chief factors of his recovery. The cases of the consolidation of the lungs recovering are also unusual, and show that even here, with the amount of waste that ensues during the career of the disease, old age is at times able to combat with it. In such cases I find that one ounce of equal parts of brandy and milk, injected per rectum every two hours, produces most satisfactory results. I would make an observation regarding the diet. I always endeavour, as far as possible, to give the patient the same kind of food which he or she is accustomed to in health; and if I find that the stomach rebels against that, to give it as little trouble as I possibly can, and resort to nutrient enemas, given in small quantities and at frequent intervals. In summer or winter I invariably have a hot-water jar placed in the bed, as the lessened muscular energy, with consequent diminution of animal heat in old age, is considerably increased by the disease. These, perhaps, are small things; but even they are worthy of our notice and consideration.

